# Systematically reviewing and synthesizing evidence from conversation analytic and related discursive research to inform healthcare communication practice and policy: an illustrated guide

**DOI:** 10.1186/1471-2288-13-69

**Published:** 2013-05-30

**Authors:** Ruth H Parry, Victoria Land

**Affiliations:** 1 Sue Ryder Centre for the Study of Supportive, Palliative and End of Life Care, University of Nottingham, Queens Medical Centre, Nottingham, NG7 2HA, UK; 2 Independent Researcher, York, UK

**Keywords:** Systematic review, Conversation analysis, Discourse analysis, Healthcare, Quality appraisal, Methodology

## Abstract

**Background:**

Healthcare delivery is largely accomplished in and through conversations between people, and healthcare quality and effectiveness depend enormously upon the communication practices employed within these conversations. An important body of evidence about these practices has been generated by conversation analysis and related discourse analytic approaches, but there has been very little systematic reviewing of this evidence.

**Methods:**

We developed an approach to reviewing evidence from conversation analytic and related discursive research through the following procedures:

• reviewing existing systematic review methods and our own prior experience of applying these

• clarifying distinctive features of conversation analytic and related discursive work which must be taken into account when reviewing

• holding discussions within a review advisory team that included members with expertise in healthcare research, conversation analytic research, and systematic reviewing

• attempting and then refining procedures through conducting an actual review which examined evidence about how people talk about difficult future issues including illness progression and dying

**Results:**

We produced a step-by-step guide which we describe here in terms of eight stages, and which we illustrate from our ‘Review of Future Talk’. The guide incorporates both established procedures for systematic reviewing, and new techniques designed for working with conversation analytic evidence.

**Conclusions:**

The guide is designed to inform systematic reviews of conversation analytic and related discursive evidence on specific domains and topics. Whilst we designed it for reviews that aim at informing healthcare practice and policy, it is flexible and could be used for reviews with other aims, for instance those aiming to underpin research programmes and projects. We advocate systematically reviewing conversation analytic and related discursive findings using this approach in order to translate them into a form that is credible and useful to healthcare practitioners, educators and policy-makers.

## Background

Our objective in this paper is to describe a step-by-step guide aimed at those wishing to systematically review conversation analytic and related discourse analytic evidence on relatively specific topics or domains (we are not writing here about the methodology of doing either conversation or discourse analysis). The guide is particularly tailored to reviews where the aim is to inform healthcare practice and policy. Throughout, we draw on a review we conducted to examine evidence about how people talk about sensitive future matters, including illness progression, death and dying [1].

We first provide some background, briefly outlining the rationale and core procedures of systematic reviewing, then providing an overview of the value and methods of conversation analytic and related discursive research. We then present our step-by-step guide. In describing the stages, we examine some distinctive features of conversation analytic and related discursive research which must be addressed when conducting reviews and which mean that established review procedures need combination, alteration, and adaptation for systematic reviews of conversation analytic and related discursive evidence.

### Systematic review and synthesis

The rationale and development of systematic review methods have been extensively discussed [2-6]. In brief, the overall purpose is to sum up best available research evidence in relation to a specific question. The process entails employing recognised and replicable procedures to find, evaluate, and draw together the findings of relevant research. Whilst any reviewer of the literature might well aim to be systematic in their reviewing, the term systematic review is used to mean a specific approach. Compared to traditional (or informal) literature reviews and summaries, systematic reviews aim to be more comprehensive, formalised and transparent, and less dependent upon individual reviewers’ interests – interests which can open traditional summaries to bias [7]. In the field of healthcare research and practice, the findings of this kind of review are seen as more credible than other forms of literature review [8].

Procedures followed in systematically reviewing and synthesizing quantitative evidence are well-established [4]. For qualitative research, methods are rather more diverse and contested [2,9,10]. However quantitative and qualitative review approaches share some core procedures. After formulating the review question(s) and scope, extensive searches for evidence are conducted, often with a particular emphasis on electronic databases, using sets of keywords to interrogate these. There follows a progressive sifting of identified publications by applying explicit inclusion and exclusion criteria, then an appraisal of quality using a ranking tool and/or checklist. For publications included in the final set, characteristics of the studies they report - such as the design and participants, and details of the findings - are ‘extracted', that is, summarised and recorded using standardised forms. The final stages involve synthesis of the evidence - comparing and integrating findings, and consulting extensively with interested parties so as to draw conclusions and formulate explicit recommendations [11,12]. For quantitative research, synthesis, i.e. combining the findings of multiple studies, usually involves applying statistical procedures. For qualitative research, an increasingly diverse range of approaches exists for combining findings of multiple studies [2,6,10,13]; these can be understood as falling into two broad sets of approaches [2]. One set, which has been termed ‘aggregative synthesis’ [9], entails a focus on describing and summarising findings ‘(often in a highly structured and detailed way) and translating the studies into one another.’ ([2], p 8/11). The other set of approaches can be termed interpretive syntheses [9]; these ‘seek to push beyond the original data to a fresh interpretation of the phenomena under review’ ([2], p 8/11), and their ‘primary concern is with the development of concepts and theories that integrate those concepts.’ ([9], p 2/13).

### Conversation analysis

The vast majority of healthcare delivery - from diagnosis to decision-making, and from implementing procedures to measuring their effects - is accomplished in and through conversations between people. The quality and effectiveness of healthcare depend enormously upon how people manage these conversations, and thus on the communication practices employed within them [14,15]. Over the past four decades, and particularly in the last fifteen years, great strides have been taken in scientific understandings of human communication practices and behaviours – particularly those derived from naturalistic observations of large numbers of communication episodes [16-18] rather than from experiments or qualitative interview studies. A substantial contribution to this progress has been made through conversation analytic investigations. Despite the name, conversation analysis is applicable in any setting where people interact, including: family conversations [19,20]; consultations with doctors [18], nurses [21], psychotherapists [22], and physiotherapists [23]; surgical procedures [24,25]; and interactions in legal [26], mediation [27,28], and social support settings [29,30].

Although many conversation analytic studies have collected and analysed data from health and social care settings, the approach has been developed, applied and published largely by those working in the academic domains of linguistics, sociology and social psychology. It is currently less familiar to those working in medical and health services research, amongst whom the term discourse analysis is somewhat more familiar than conversation analysis. Discourse analysis is an ‘umbrella term’ that encompasses a wide range of approaches to analysing texts and talk [31]. In contrast, conversation analysis is a single, specific, defined, and bounded research approach with an established set of perspectives and methods [32]. Some discourse analytic approaches share areas of common ground with conversation analysis [33] and the review methods we describe allow for this kind of discourse analytic work to be incorporated into a review. However, in order to avoid cumbersome wording, hereafter we use only the term conversation analysis.

Conversation analytic studies rely on audio and, increasingly, audio-visual recordings of interactions between people. Recording is planned and conducted so as to minimize the intrusiveness and effects of recording on behaviour [34-36], aiming to capture what would go on whether or not the research were in progress [31]. Whilst it is impossible to prove definitively that data captured reflect what would have occurred had recording equipment not been present [37], there are good reasons to assume data is valid in important respects [36]. Recordings are subjected to repeated listening and viewing, and collections of the phenomenon/a of interest are made. For instance, when investigating healthcare, collections might entail episodes where bad news is delivered [38]; where the topic of alcohol or smoking is raised [39]; where patients resist a treatment proposal [40]; or where consultations get brought to a close [41]. Collected episodes are closely scrutinized to generate descriptions of typical and atypical features of communication sequences. These features include: who does what and in what order; what phrases and words are used, and what body movement patterns can be observed. Episodes are transcribed using established conventions [42] which include information about pacing, intonation and overlapping speech, as well as the words used. Analysis draws heavily on previously established findings about communication practices and their functioning [43]. Once practices and patterns of communication have been identified and described in close detail by reference to specific (and often numerous) data sequences, empirical findings are used to generate understandings about the functioning and outcomes of particular practices.

Whilst there have been some literature reviews examining conversation analytic evidence in relation to specific phenomena and domains [44,45], to the best of our knowledge only one systematic review has been published [7]. This pilot review by Nowak examined German language research on doctor patient talk. Whilst drawing upon a number of approaches to synthesizing qualitative research, Nowak’s review was ‘largely designed in accordance with the research process of the “meta-narrative review”’(p. 430) - a pre-existing *off the shelf* review approach. Whilst we too draw considerably on existing systematic review procedures, we propose that no pre-existing off the shelf approach is adequate for handling conversation analytic evidence. Thus in the review we conducted, whilst we drew extensively on components of existing review approaches, we also developed new components fitted to the distinctive features of conversation analytic work for which existing quantitative and qualitative review approaches could not provide a solution. Also, Nowak’s systematic review [7] involved generating ‘new theoretical concepts’ (p430, see also p436) within the synthesis phase by using a grounded theory approach. Our approach does not involve use of interpretive processes to develop new theoretical concepts, but entails aggregating findings so as to draw out clinical, policy and/or educational implications.

The significant knowledge conversation analytic studies have generated about verbal and embodied communication practices and their consequences has been little accessed and recognized in healthcare policy, education and practice. This reflects the fact that many studies have been framed in terms of sociological and linguistic concerns, theories and debates, and reported in sociological and linguistic publications. The evidence thus remains largely confined within its parent academic fields. Our paper is motivated by a conviction that this knowledge should no longer remain unavailable to clinical practice and education. Systematically reviewing this kind of evidence is particularly timely because conversation analytic findings are increasingly being used to underpin quantitative evaluation [46], communication training [47], and interventions which have proven effective in enhancing health and social care practice [48-50].

### Background to the review of future talk

We conducted a review of evidence about how people talk about sensitive and uncertain future matters including illness progression, dying and death. The review protocol can be found at the PROSPERO website [51], an initial summary of findings is reported elsewhere [1], and a more extensive report is in preparation. The work was initiated in a context of growing debate and policies proposing that members of the public [52] and healthcare professionals [53,54] should talk more than they do about individuals’ death and dying, and that this should lead on to explicit planning for end of life care. At the same time, it is clear that both public [55] and professionals [56] find broaching this topic difficult, and patients and families report very unsatisfactory experiences [57]. Some of the review team knew of conversation analytic studies investigating how people talk about these sensitive topics and documenting the consequences of different ways of talking about them in settings including HIV counselling [58] and oncology clinics [59]. We also knew these had largely been reported in sociology and linguistics publications. We concluded that drawing together evidence in this area would enable us to generate useful, practice-relevant information. We recognised that applying a systematic review approach would enhance the likelihood that findings would be seen as credible by our intended audiences.

## Methods

In order to develop our approach, we reviewed methodological reports, reviews, and discussions of existing approaches – particularly those about systematic reviews of social scientific research and evidence [2,3,9,10,12]. We then drafted an outline plan for the proposed steps in our review by drawing upon both this existing literature and the review team’s and advisory group members’ expertise in systematically reviewing quantitative, mixed methods and qualitative research [60-64] and in conversation analysis [65-67]. We discussed and reached consensus on these proposals with our review advisory group. An iterative process followed in which trying out, reflecting upon, and refining methods for each stage of the review culminated in the guide we present here.

In the following sections, we describe our review approach in terms of eight stages. The approach is tailor-made for working with conversation analytic and related discursive evidence, and we illustrate from our ‘Review of Future Talk’. The stages vary in the degree to which they are based upon and borrow from established and previously reported review practices. For those that are similar, we cite original sources; for those that are dissimilar, we provide detailed explanation, description and some additional files containing various templates. In discussion, we reflect on the challenges and value of systematically reviewing this kind of evidence, and note some possible adaptations and developments of our approach.

## Findings: step-by-step guide

Table 1 summarises the eight stages of reviewing. Our proposals should be treated not as rules but as guidelines to be applied flexibly to individual cases. Despite the linear layout of our table and description, in reality the process involves considerable overlap and looping between stages. A note on managing the process: reviews require handling large amounts of data and performing various operations on that data, and may also involve geographically spread teams. Technologies that allow teams to organise the data and communicate efficiently include online reference management software and online file storage. Thus in our review, we maintained a review record in electronic document form. Each reviewer completed and revised sections, and consecutively numbered versions as they added to the record. We shared these and other files via an online file storage programme [68]. Electronic database searches were downloaded to online reference management software [69] which allowed checking for and removal of duplicates, and maintenance of different folders for original searches, and for included and excluded papers. Email discussions, phone conversations, and face to face meetings were also important elements of the process.

### Stage 1: Articulate purpose and audiences, then articulate review question and scope

In explicitly articulating the purpose of the review, including its intended audience(s), reviewers build the essential foundations for subsequent deliberations about the review question(s) and scope, and for making decisions about the relevance of individual papers and specific bodies of work. In terms of process, defining purpose and audiences requires reading and deliberation within the review team, and consultations with a range of people with relevant expertise and insights, including practitioners and academics. These consultations comprise face-to-face discussions and circulation and revision of drafts. Only once purpose and audiences are clear should reviewers begin to formulate the review question(s) and scope.

In the Review of Future Talk, deliberation and consultation led to the following definition of the review purpose: “*To inform healthcare practice, policy and training with regards providing opportunities for communication about sensitive future matters, including death, dying and planning for end of life*”. The phrase ‘*with regards providing opportunities for communication about*….’ articulates an agnostic stance towards the rights or wrongs of providing such opportunities, and was incorporated as a result of both clinical and conversation analytic perspectives expressed during consultations. The review purpose remained unchanged throughout and provided an anchor point of certainty amidst the sometimes perplexing task of deliberating about whether particular bodies of work and individual publications should be included.

The next step involves articulating the review question(s) and the scope. Defining scope means deciding as precisely as possible which communication practices and tasks, and which conversational participants and settings, will be treated as relevant. This is not easy because communication practices, tasks and activities are not neatly demarcated, and they do not fall into mutually exclusive categories. People generally do more than one thing at the same time through their communication; and any particular communicative task can be attempted and accomplished via multiple practices: think, for instance, of the multiple ways in which one can attempt to ascertain information, including asking direct questions; issuing ‘fishing’ comments; conveying confusion; and raising concerns. (An academic discussion bearing on

**Table 1:** Stages of systematically reviewing and synthesizing evidence from conversation analytic and related discursive studies

	**Stage**	**Description of the process**
**1**	**Articulate purpose and audiences, then review question(s) and scope**	Discuss then articulate the review’s purpose and the audience(s) for its findings
		Articulate review question(s) and scope – define the topic, phenomenon or domain of interest through engagement with literature and deliberative discussions
**2**	**Specify eligibility criteria**	Some criteria apply to all reviews:
		Studies must rely on fine-grained analysis of audio / audio-visually recorded naturalistic interaction
		Not only interactional data but also its analysis pay explicit attention to the topic, phenomenon or domain of interest
		Devise other criteria, including about settings and language, according to needs of the individual review
**3**	**Search for studies**	Identify potential sources of publications including electronic databases, specialist bibliographies, and knowledge amongst the review team and its contacts
		Design, test and refine word groups for database searches
		Search sources and record results
		Scan identified publications, make inclusion decisions based on eligibility criteria and definition of scope
		For difficult cases, read in detail and discuss within the review team to make decisions
**4**	**Describe characteristics of included studies**	Unidimensional quality appraisal is not possible for this kind of evidence, instead record characteristics of data, settings, participants, analytic approach, and analytic depth in order to specify studies’ contribution to the review
		Design customised templates for collecting this information
**5**	**Data extraction**	Design customised data extraction template
		Complete extraction for each study
		Collect relevant data extracts from each study
**6**	**Collate and synthesise data**	Read completed data extraction forms
		Organise studies into logical categories
		Organise and combine findings into logical categories
		Consult wider literature in relation to practices identified
		Consult with end users
		Identify gaps in the evidence
		Derive implications for the review audience(s)
**7**	**Sensitivity and subgroup analyses**	Retrospectively assess the contribution of different sets of findings or sets of publications to the review
**8**	**Reporting**	Consult review advisors and representatives of intended audiences
		Draw on established guidance for the reporting of systematic reviews (including 'PRISMA' guidelines)
		Include tables summarizing study characteristics and study findings

 this point can be found in those sections of Levinson’s “Pragmatics” text which examine the ‘Literal Force Hypothesis’ [70]). Furthermore, by their very nature, communication practices and tasks do not carry explicit or self-evident ‘labels’. For these reasons, finalising the questions and defining the scope for a conversation analytic review is a lengthy process. In practice it involves initial searches for and reading of potentially relevant publications, and discussions between reviewers and advisors. This is similar to processes used in established approaches for reviews on complex topics [9,12].

In the Review of Future Talk, the review questions and scope were redefined and specified with increasing precision over the first six months of the two year project. The resulting primary question was: ‘*What evidence exists about how people initiate and pursue talk about sensitive future matters including death, dying and planning for end of life?.*’ Defining the scope (see below) required reaching clarity about what would count as ‘*sensitive future matters*’ for the purposes of the review. Some aspects were clear: studies about talk on future matters that were not directly personal (e.g. talk about global climate change) and studies examining talk about future positive achievements were ruled out by the review’s purpose of informing a particular area of healthcare practice. However, others were less clear: for instance what we meant by ‘sensitive', and whether to include studies that examined people’s talk about the future in relation to *currently existing* troubles.

### In the Review of Future Talk, the final definition of the scope in terms of 'talk about sensitive future matters' was as follows

For the purpose of this review we define talk about sensitive future matters as talk where there is reference to states, events and/or actions:

•In the domain of individual persons (rather than, e.g. the Earth’s climate)

•Spanning those that are uncertain to certain, contingent or not

•That may or will happen in relation to individual persons, and are oriented to - or orientable to - in the specific context as negative or as having potential negative implication(s)

•That may or will happen some time after the current interactional episode

We include:

•Studies where talk about future sensitive matters is inherent to the activity examined in the research, and also those where it is adjunctive and occasional

•Studies of talk about future sensitive matters whether or not talk includes or aims at making plans or decisions about future actions in relation to individuals’ care and lives

We do not include:

•Studies where analysis examines talk that is exclusively focused on possible future actions in relation to currently existing troubles (as is found in many studies of advice giving)

### Stage 2: Specify eligibility criteria

Eligibility criteria specify *a priori* which kinds of evidence will be included in a systematic review. In quantitative reviews, criteria are generally narrow [4], with only certain study designs eligible for inclusion e.g. randomised controlled trials. Similarly, reviews of conversation analytic evidence should be restricted to studies that rely on detailed inductive analysis of audio- or audio-visually recorded naturalistic interactions. Studies where recorded naturalistic data are analysed solely or primarily using coding frameworks are excluded. Furthermore, to be relevant, studies must include not only interactional data but also analysis that explicitly attends to the topic or phenomenon of interest. Because of the richness and complexity of communication, it is common - and rather frustrating - to find publications where data extracts show participants directly engaging with the matters that are of interest to the reviewers, but where the analytic focus of the publication itself is on other matters. Commonly in systematic reviews, limits are set in terms of how long ago evidence was published. In our view, given the cumulative nature of conversation analytic research, the fact that the term conversation analysis was not used before the 1970s, and the relative stability of communication behaviours, it is logical to include publications from any date in reviews of conversation analytic evidence. Other eligibility criteria should be defined for individual reviews; considerations should include: whether or not to exclude studies outside healthcare; whether to restrict to studies analysing data from only one language; and whether to include unpublished studies such as graduate theses.

In the Review of Future Talk, we included studies of talk about future sensitive matters whether the setting was formal and institutional (e.g. health or social care episodes) or informal (e.g. friend and family conversations). This decision was consistent with the conversation analytic view that practices used in institutional interactions are grounded in, rather than distinct from, everyday communication practices [71]. We did, however, exclude studies of large-group interactions, such as classrooms, as these are so different to healthcare consultations which usually involve just two or three people. We excluded studies where data involved languages other than English because of the possibility that different languages might entail significantly different practices for talking about the future, and/or different consequences of practices. We did, however, keep copies and notes on non-English studies that we identified. This allowed us to make preliminary observations about whether practices identified in the main review had been identified in other languages. We included only studies published in peer-reviewed journals or published books, and excluded conference presentations and graduate theses. In so doing, we treated the peer review process as a form of quality control upon the publications included in our review (although we acknowledge that, like any quality control, peer review is not without flaws).

### Stage 3: Search for studies

#### 3a) Identify potential sources of publications, search sources

As noted above, many conversation analytic investigations relevant to healthcare are published outside clinical journals and in disparate fields including linguistics and sociology. For this reason, diverse sources need to be searched. Doing so is established practice in systematic reviews of complex interventions and those where social science literature is examined [3,72]. Therefore, besides interrogating electronic databases using standardised sets of search terms, other sources are used. These include the review team’s existing knowledge, and knowledge amongst the conversation analytic and academic healthcare community accessed via personal contacts, forums such as electronic discussion lists, and online bibliographies. ‘Snowball sampling’ – i.e. citation tracking and reference searching of publications identified through these various means should also be used. With regards formulating search terms for use with electronic databases, the services of a librarian/information specialist should be sought if possible ([4], Section 6.3.1).

In the Review of Future Talk, we tested and refined sets of ‘word groups’ in order to maximize sensitivity and specificity of the electronic database searching. Terms that we found most useful in identifying studies that applied conversation analytic and related discursive methodologies to our substantive topic were: (Group 1) communicat* OR interact* AND (Group 2) audio* OR video* OR discourse-analysis OR conversation-analysis OR sequential-analysis OR linguistic*. Details of all the word groups we used can be found in Additional file 1. We also searched for publications from sources including our own Endnote databases, the bibliography section of the ‘Ethno/CA News’ website [73], and an enquiry to the ‘Languse’ internet discussion list [74]. Once we had identified papers from these sources, we searched for potentially relevant papers amongst citations of these using the ‘Google Scholar’ database [75]. At the time of the review, we did not have the resources to call upon the services of an information specialist. Whilst this may have resulted in a less than optimal search strategy, we believe it did not have a huge impact on our review because, as we explain below, five out of the 18 papers we eventually included were not listed in any of the multiple electronic databases we searched.

#### 3b) Scan identified publications and make final selection for inclusion

Each round of searching usually identifies a relatively large number of publications compared to the number finally included. Identified publications need sifting to decide which fall within the review’s scope and eligibility criteria. This can usually be judged merely by examining title and abstract, and whilst established guidance states that it is desirable for two reviewers to do so ([4] Section 7.2.4), for reasons of practicality it is not uncommon for just one reviewer to perform this initial sifting [76]. Where decisions cannot be made from title and abstract alone, the full paper must be obtained and the data extracts and analysis sections read closely. At this point, for the sake of reliability, it is ideal practice for two reviewers to undertake reading and judgements separately. Even after closely reading extracts and analysis, there are often boundary cases for which decisions about inclusion are not straightforward. After these have been read by at least two members of the team, they should be discussed in order to reach reasoned consensus decisions about inclusion. Where a publication has been read and excluded, notes should be kept on the decision made and the reasoning behind the exclusion as this helps later report writing, and expedites any process of revisiting or even revising decisions.

In the Review of Future Talk, we identified over 2000 publications through our broad search strategy. Eighteen publications were included in the final review. We opted to search nine different electronic databases (ISI Web of Science, Amed, Embase, CINAHL, Medline, PsycINFO, ASSIA, Sociological Abstracts CSA, Google Scholar) because we were interested in whether any would stand out as particularly useful or not for conversation analytic publications. The least useful databases for us in terms of the proportion of publications identified to those actually included were: (a) PsycINFO where searching identified 844 publications, only three of which were finally included and two of those were also found in other databases; (b) Sociological Abstracts where searching identified 284 papers, none of which were included in the final review. We found the ISI, Embase and Medline databases produced fewer ‘false positives’ - each yielding fewer than 160 ‘hits’; three publications which were found in these databases and not found from any other source were included in the final review. Notably, ten of the finally included papers were not identified in any of our electronic searches. After completing our review, we checked back and found that five of these ten were listed in the electronic databases, but had not been identified in our searches, and that the other five were not listed in any of the databases.

Of the final 18 publications we included, four were found exclusively from electronic database searching, 10 were found through reviewing our existing knowledge, one was a serendipitous find, and the other three were each identified twice – both in the databases and via our existing knowledge. Eight of the 18 were listed in the 2011 version of the specialist ‘EMCA news’ specialist bibliography [73]. In our discussion, we consider the pros and cons of searching various sources, particularly electronic databases, for this kind of review.

In the searching and sifting stage, we found 15 publications for which it was not possible to make definitive inclusion or exclusion decisions without detailed reading. Each of the two main reviewers read and then discussed them in order to reach consensus decisions. Five of these 15 were included in the final 18. Our discussions about these ‘boundary cases’ and our notes on reasons for exclusions and inclusions were important in reaching a final version of the review’s scope.

### Stage 4: Describe characteristics of - rather than appraise - included studies

Existing techniques, guidance and discussion about appraising the quality of quantitative ([4], Chapter 8) and qualitative [77,78] research have very limited application to conversation analytic research for the important reason that conversation analytic perspectives, methods and findings are incompatible with the binary categories - qualitative and quantitative - familiar in healthcare research [43]. The primary data and findings of conversation analyses are not numerical and statistical (although studies increasingly include tabulations and descriptive statistics as part of their findings [71,79,80]) so conversation analytic work does not fall within the scope of quantitative healthcare-related research. The conversation analytic approach is also incompatible with conventional understandings of qualitative enquiry as entailing investigating meanings, views and understandings via interpretive analysis, most commonly using transcribed interview data [81-83]. In conversation analysis, the main data always comprise directly recorded interactions rather than qualitative interviews, and conversation analysts explicitly and strictly avoid using data to impute psychological states, perceptions and motivations [84,85]. Conversation analysis produces systematic and empirically grounded descriptions of concrete practices and their interactional consequences and functioning, it does not involve the kind of interpretation and theory generation that characterise in qualitative healthcare research [84]. These distinctive features mean that no existing tools for quality appraisal of research are suitable.

So, what can be done in terms of characterising the contribution made by each conversation analytic study included in a systematic review? Rather than reaching a single assessment of each study’s quality, or ranking studies, two broad dimensions must be considered in relation to each study’s value and contribution: (1) the type and amount of data, and (2) the detail and depth of analysis. These two cannot be collapsed into a meaningful, single, quality assessment. *The type and amount of data*: conversation analytic studies vary with regards whether audio or audio-visual recordings are used, what amount of data is analysed, how many settings and participants are involved, and how diverse is the range of settings and participants. It is inappropriate to assume that more data is better: studies that document practices in substantial detail regularly involve quite small datasets. Also, one type of conversation analytic research involves bringing the cumulative findings of past work about the use and functioning of interactional practices to bear upon single episodes of interaction [86]. On the other hand, some studies examining larger datasets examine practices in less detail, but may significantly contribute to reviews by providing evidence about how widespread a practice is, its frequency of use within settings, and by showing recurrent patterns in its consequences such as the kinds of response it prompts from patients. *The detail and depth of analysis*: studies vary greatly in the detail and depth to which they analyse particular interactional practices or phenomena. Variations include the degree to which they examine when in their interactions (and communication sequences) people use some particular practice(s); and whether or not they examine only the words used or other important language features such as grammar, pauses, and intonation. They also vary in the degree to which they investigate the consequences and/or social functioning of the practice. If analysis examines few of these features, this is not necessarily equivalent to lower quality – studies often explicitly set out to examine restricted aspects of a phenomenon, practice or domain in great depth. Studies also vary in terms of the extent to which analysis is grounded in previous empirical findings. Again, it is not logical to treat this as a simple matter of analytic quality, because it is impossible for earlier studies to refer to later findings.

Thus, reviewers should not claim that studies with more analytic detail, depth and grounding provide stronger evidence, nor that studies documenting the practices or phenomena among more numerous or diverse people and settings provide stronger evidence. Rather, studies documenting a practice ‘more widely’ contribute one type of evidence, while those documenting a practice ‘more deeply’ contribute another type of evidence. Rather than applying conventional quality appraisal tools, conversation analytic reviewers must collect and present information on several dimensions of the studies. Any proposals concerning the strength of evidence about particular practices or phenomena need to be described and justified in terms of these various dimensions. Reviewers need to record these details using a customised template designed to capture characteristics of each study, its dataset, and its analysis.

The characteristics we recorded for studies in the Review of Future Talk are listed in the subsection below. Additional file 2 provides a formatted version of the template we used.

#### Characteristics recorded for studies in the Review of Future Talk

Data characteristics:

•Size of overall dataset in minutes / hours, and number of interactions

•Number of episodes from the overall dataset upon which analysis relies

•Number of episodes from the collection that appear in the publication

•Number and description of sites

•Number and description of institutional contexts (e.g. hospital ward, outpatient clinic, family conversation)

•Whether practice(s) is/are observed in more than one individual/dyad

•Whether practice(s) is/are observed in more than one group (e.g. do both doctors and patients use it)

Analysis characteristics: Does analysis:

•Predominantly examine more than only one party’s turns; i.e. attend to sequence?

•Examine data in fine-grained detail?

•Examine more than just the topical/semantic content; i.e. does it attend to aspects of grammatical, pragmatic, and/or prosodic content?

•Include examination of aspects of the sequential environment in which practice(s) occur(s)?

•Include examination of aspects of turn and/or sequence design?

•Include examination of interactional effects and consequences?

•Include examination of atypical cases?

•Support central analytic claims by direct references to data/extracts?

•Use established analytic findings as ‘tools’ in the analysis?

### Stage 5: Data extraction

Data extraction is the term conventionally used in systematic reviewing for the work of recording findings, claims, and data from each included publication [3,4]. Besides recording findings, ‘facesheet’ data are gathered – these document basic details such as study title, date of publication, authors, and journal or book title. For recording findings, reviews of conversation analytic evidence require customised templates to collect information on the kinds of phenomena and analytic dimensions that conversation analytic studies report, and they also need to handle the fact that studies often describe more than one practice or phenomenon. Development of templates should include blind testing on a diversity sample of the included papers, with two or more reviewers completing templates for several papers independently, and then comparing results. This allows ambiguities and missing elements to be identified and then resolved in subsequent drafts. The subsection below lists the analytic dimensions recorded for each paper in the Review of Future Talk. A version of our data extraction template can be found in Additional file 3. Another set of data is also collected: original data extracts from each publication. Where only part of a publication’s findings are relevant to the review, only the associated data extracts are collected. It is worth noting that extracts comprise edited sections of transcripts rather than the original data, so cannot be used for the purpose of going beyond existing evidence to build new findings; rather they are used to support the collation and synthesis of the findings, and for illustrating reports.

#### Data extraction categories used in the Review of Future Talk

•Phenomenon (in brief)

•Phenomenon in author’s own words

•Research question for this finding (if applicable)

•Number of episodes pertaining to this finding

•Archetypal sequence

•Features of the talk in which the phenomenon is produced – i.e. aspects of the sequential/interactional context in which it arises

•What are the implications of these environmental features?

•Sequence and/or turn design features of the phenomenon

•What are the interactional effects of these design features?

•In sum, what is the overarching function of the phenomenon?

•Author-proposed implications

•Any other implications

•Reviewer’s notes

### Stage 6: Collate and synthesise data

At this analytic stage the studies are organized into logical categories [3]. There is no definitive or ‘correct’ organisation, rather the process must be driven by the review’s purpose and questions. An obvious way to organise conversation analytic evidence is in terms of the practices documented; other options include organizing according to study setting or the kinds of participants recorded. Next, findings are analysed within each category so as to combine understandings about particular practices. Tables summarising the characteristics and findings of the included studies are compiled.

Synthesis begins with an overall description of the amount of information uncovered through the review [3]. Findings are then collated and summarised using an aggregative approach – drawing together findings in ways that involve describing, summarising and what has been termed ‘translating into’ each other [2]. At this stage, reviewers of conversation analytic evidence may draw upon, and indeed systematically search for, other literature in order to expand the insights provided by the review. Doing so is established practice in review approaches for evaluating complex interventions [12]. Synthesis also involves identifying gaps in the evidence. This requires understanding what is actually done and required in practice, and comparing this with those communication practices and actions that have been investigated and documented by research. Synthesis culminates in generation of evidence-based, reasoned proposals about the implications for the review’s audience(s).

A specific and distinctive feature of conversation analytic findings must be dealt with when synthesizing the evidence and formulating explicit recommendations: conversation analyses find consistently that there are always multiple ways to perform any communicative activity, with each way having an array of advantages and disadvantages for any individual context and endeavour. It is therefore not possible to produce conclusions such as: ‘Practice X should be used, and practice Y should not’; or ‘X works, and Y does not’. Such blanket recommendations are incompatible with a scientific approach that takes seriously the complexity of human communication and the way that communication practices are always fitted to individual contexts and interlocutors. Reasoned proposals or implications generated in reviews of conversation analytic evidence thus need to take a form similar to that recommended within an existing approach called explanatory review (sometimes known as realist synthesis) [12], along the lines of: “In circumstances such as A, try practice B, or when using C, watch out for D” ([12], p S1:24). The proposals should be tested, extended and refined by reporting them to and discussing them with relevant audiences and experts before they are finalised.

In the Review of Future Talk, we organised the findings in terms of practices. These included: ‘agenda setting questions’; ‘use of hypotheticals’; ‘allusive, vague, or euphemistic talk’; and ‘features other than words that display sensitivity’. We then considered two alternative ways of ordering these categories for reporting. One was to order them according to how much evidence there was about each – in terms of both analytic detail and depth, and ‘quantity/diversity’ of data, participants and settings in which the practice had been observed. The alternative was to mirror the interactional sequences we were interested in, that is, to start with practices used in attempting to initiate talk about sensitive future matters, then report on those used in pursuing such talk, and finally those that closed talk about these matters. Given that the primary purpose of our review was to provide practice, education and policy relevant information (rather than, for instance, to set a research agenda), we decided this latter ordering would be the most helpful for our intended audiences. As we moved to synthesis, we drew on seminal and recent studies and reviews in order to strengthen findings and extend the usefulness of the review. Specifically, we used these to add information about how and why particular practices had particular effects on encouraging or discouraging talk about future sensitive matters. For instance, work on how questions function within medical interactions [44] offered additional insights into the mechanisms through which talk about future troubles is encouraged by the question-asking practices identified in our review. However, as noted, we did not aim to generate new findings or theories on the basis of our review or through re-analysis of data extracts; in this sense, the synthesis approach we used was aggregative rather than interpretive.

### Stage 7: Sensitivity and subgroup analyses

Conventionally, sensitivity analysis involves assessing *post hoc* the effects of including or excluding particular findings [9]. Subgroup analyses examine whether the findings vary in relation to particular characteristics of included studies (or their participants) [87]. Subgroup analyses can be used to examine the effects of including studies yielded from particular sources, and so inform design of search strategies for subsequent reviews.

As noted, only three publications included in the Review of Future Talk were identified exclusively through electronic database searching. We decided to perform a subgroup analysis examining the usefulness of particular sources for finding publications in this kind of review. We examined the three publications that had been identified exclusively through electronic database searching – those that had not been found via any source. We found that one of these documented a practice about which more extensive evidence was provided by other publications, and that the other two involved analysis that examined wording but not other important features of communication, and which focused largely on clinicians’ talk rather than including examination of patients’ responses. On the other hand, all three of these studies documented the practices across relatively large numbers of cases. The value to the review of these three studies was in adding to the extent of evidence, rather than adding details about practices’ structure and functioning. We concluded that in our particular review we would have drawn the same conclusions in terms of implications had we not included these three publications (i.e. had we not searched electronic databases), but that on the other hand, these three strengthened the credibility of the review because they contributed evidence that the identified practices are widespread in their use.

### Stage 8: Reporting the review

Reviews should be reported in a form that is accessible, useful and credible to the audiences for whom it is designed. Consulting with potential users and asking them for comments on draft versions is thus an important element of reporting. The kinds of applied and clinical journal to which reports are likely to be submitted often impose strict word length limitations. It is thus useful to make additional information such as reproductions of extracts from included papers available e.g. through an extended version published on the journal’s website. Most if not all clinical journals will expect quantitative systematic reviews to be reported in a format laid out in the ‘PRISMA statement’ – well established guidance for reporting systematic reviews that evaluate healthcare interventions [87]. Although this guidance is specific to one type of quantitative systematic review [88], it is advisable to use some of its features in reporting reviews of conversation analytic evidence - including using flow diagrams to set out information about numbers of publications initially found, numbers sifted out, and numbers finally included. Reports should also include tables summarising as briefly as is feasible the characteristics of each study, and studies’ findings and claims. Specific guidance on reporting reviews other than the type covered by the PRISMA statement is emerging (e.g. [88]) and should be consulted in writing journal reports.

We presented and discussed emerging findings from our Review of Future Talk with several clinician and educator audiences including people with different professional backgrounds, more and less experience, and working in different settings. We also held discussions with individual colleagues before attempting to write a report for publication. This proved useful in alerting us to which elements seemed of most interest to our audiences, and to the kinds of concerns they might express. For instance, some clinicians reacted to our proposals with concerns and questions about the extent to which changing their communication behaviours would constitute manipulating patients, and we thus chose to address this concern within written manuscripts. On the other hand, our verbal presentations about findings reassured us that although most clinicians saw themselves as already having tacit knowledge of the practices we described, they found it useful rather than patronizing to have this knowledge made explicit. Presentations and discussions also gave us the opportunity to seek advice from clinical and education colleagues about our proposed implications. We were careful to make it clear that these implications were extrapolations from the data – and thus needed to be treated more tentatively than the empirical findings of the original studies.

## Conclusions

We have offered an eight-stage guide tailor-made for conducting systematic reviews of conversation analytic evidence, shaped particularly for reviews that aim to provide useful information to professionals, policy makers, and educators. The process supports systematic location, collation and examination of evidence derived from conversation analytic and related discursive work. It could be adapted for reviews with other aims, such as literature reviews where the aim is inform research agendas, or to underpin doctoral theses. A possible adaptation of the approach would be for reviews to include re-analysis or secondary analysis of original data, along the lines of meta-analysis in quantitative reviews. Whilst theoretically this could be a very fruitful means of extending conversation analytic knowledge, there are practical problems particularly in relation to evidence about healthcare because of the restrictions that usually (and appropriately) exist in terms of sharing data.

Systematic review work is time consuming and laborious. Systematically searching for evidence – particularly via electronic databases – produces a low yield of eligible publications relative to the time and effort involved. Despite the limited contribution of publications identified this way in our own review, we nevertheless argue that it is worth spending the time required for two reasons. Firstly, this kind of process both counteracts the natural tendency for reviewers to focus only upon publications of which they are already aware and helps ensure searching of the breadth of academic fields in which conversation analytic work is published. Secondly, the systematic review process has an established reputation and credibility in applied academic fields such as healthcare [4], education [6] and social care [89]. This means that using a systematic review approach is likely to maximize the chances of conversation analytic evidence making its way into applied fields, and of being seen as credible amongst those who wield influence in the fields of healthcare practice, policy and education. It is also worth noting that although in our particular review electronic searching yielded relatively little evidence, this would not necessarily be the case in reviews of conversation analytic evidence on other topics.

Conversation analytic and related discursive studies have generated a significant, substantial and cumulative body of knowledge about healthcare communication. This knowledge is little accessed by practitioners, educators and policy makers. Systematically reviewing evidence from this form of study offers the prospect of making useful knowledge available to practitioners, educators and policy makers in a credible form. However, there are distinctive challenges in reviewing this kind of evidence. These can be managed by applying the approach to reviewing which we have presented here.

## Competing interests

RP and VL declare we have no financial or non-financial competing interests.

## Authors’ contributions

RP devised and led design of the review approach and the ‘Review of Future Talk’, supervised its execution, and drafted the manuscript. VL participated in the design and execution of the Review – including the bulk of database searching, organising data, designing templates and extracting data, and contributed to the manuscript. Both authors read and approved the final manuscript.

## Pre-publication history

The pre-publication history for this paper can be accessed here:

http://www.biomedcentral.com/1471-2288/13/69/prepub

## Supplementary Material

Additional file 1 **Word groups used for electronic database searching in the Review of Future Talk.** File containing word group terms used in searching. Click here for file

Additional file 2 **Template used for recording characteristics of data and analysis for studies in the Review of Future Talk.** File containing blank template used in recording the characteristics of each study with regards data and analysis. Click here for file

Additional file 3 **Template used for data extraction (background details, findings and claims) of papers in the Review of Future Talk.** File containing blank template used for data extraction. Click here for file
