# Supplementary material for: Systematically reviewing and synthesizing evidence from conversation analytic and related discursive research to inform healthcare communication practice and policy: an illustrated guide
Source: BMC Med Res Methodol. 2013 May 30;13:69. doi: 10.1186/1471-2288-13-69 (PMC3674894; doi:10.1186/1471-2288-13-69)
Supplement: Additional file 1 — Word groups used for electronic database searching in the Review of Future Talk. File containing word group terms used in searching. [file 1471-2288-13-69-S1.pdf]

Systematically reviewing and synthesising conversation analytic and related discursive research to inform healthcare communication practice and policy: An illustrated guide

Ruth H Parry and Victoria Land

ADDITIONAL FILE ONE:

Word groups used in electronic database searching in the Review of Future talk

Word group 1
communicat* OR interact*

Word group 2
audio* OR video* OR discourse-analysis OR conversation-analysis OR sequential-analysis OR linguistic*

Word group 3
prognos* OR palliative OR end-of-life OR decision* OR troubles

Word group 4
future

After searching on each of these word groups, results were then combined with the Boolean term AND

For the database PsycINFO, we added an additional group:
Word group 5
AND NOT biolog* OR neuro* OR gene*
