# Supplementary material for: Systematically reviewing and synthesizing evidence from conversation analytic and related discursive research to inform healthcare communication practice and policy: an illustrated guide
Source: BMC Med Res Methodol. 2013 May 30;13:69. doi: 10.1186/1471-2288-13-69 (PMC3674894; doi:10.1186/1471-2288-13-69)
Supplement: Additional file 2 — Template used for recording characteristics of data and analysis for studies in the Review of Future Talk. File containing blank template used in recording the characteristics of each study with regards data and analysis. [file 1471-2288-13-69-S2.pdf]

Systematically reviewing and synthesising conversation analytic and related discursive research to inform healthcare communication practice and policy: An illustrated guide

Ruth H Parry and Victoria Land

ADDITIONAL FILE TWO:

Template used for recording characteristics of data and analysis for studies the in the Review of Future Talk

		**Notes**
**Data**		
Overall size data collection		
Number of episodes in collection(s)	Provide the number of episodes in the collection pertaining to EACH finding	
Number of episodes from the collection that appear in the publication	Provide the number of episodes in the collection that appear in the publication for EACH finding	
Some simulation – E.g. simulated patient in interaction		
Number of sites (e.g. two sites of the same type of setting, such as 2 HIV clinics)		
Number of institutional settings		
Explicit reference to practices observed in more than one individual/dyad		
Practices observed in more than one institutional group E.g. medics AND counsellors		

**Analysis**		
Predominantly examines more than only one party's turns (i.e. attends to sequence)		
Examines more than only topical/semantic content – i.e. attention to some aspects of grammatical, pragmatic, and/or prosodic content		
Includes examination of aspects of sequential environment in which practice(s) occur(s)		
Includes examination of aspects of turn and/or sequence design		
Includes examination of interactional effects/consequences		
Includes examination of deviant cases		
Are central/key analytic claims supported by direct quote from or references to the data? NO/SOMETIMES/OFTEN		
Reviewer's judgement of degree to which analysis is fine grained NOT/MODERATELY/VERY		
Are established analytic findings used as 'tools' in the analysis? NO/OCCASIONALLY/ CONSIDERABLY		
