# Supplementary material for: Systematically reviewing and synthesizing evidence from conversation analytic and related discursive research to inform healthcare communication practice and policy: an illustrated guide
Source: BMC Med Res Methodol. 2013 May 30;13:69. doi: 10.1186/1471-2288-13-69 (PMC3674894; doi:10.1186/1471-2288-13-69)
Supplement: Additional file 3 — Template used for data extraction (background details, findings and claims) of papers in the Review of Future Talk. File containing blank template used for data extraction. [file 1471-2288-13-69-S3.pdf]

Systematically reviewing and synthesising conversation analytic and related discursive research to inform healthcare communication practice and policy: An illustrated guide

Ruth H Parry and Victoria Land

ADDITIONAL FILE THREE:

Template used for data extraction (background details, findings and claims) of papers in the Review of Future Talk

**Review details**		**Notes**
Name of reviewer		
Date of review		
Unique reference number		

**Publication details**		
Title		
Author(s)		
Year of publication		
Source of publication	E.g. Journal title, volume, issue and pp OR book reference and pp	

**Data**		
Recording – audio visual or audio only?		
Co-present participants or non co-		

present?		
Interactions are one-to-one, multiparty or both?		
Institutional or mundane interaction?		
Country in which data was collected		
Setting(s)		
Participants	Number of participants and characteristics of participants (if stated)	
Size of the data set	Include, for example, details of the number of interactions, total or average length of interactions, if multiple settings records how many interactions in each setting, and any other features of the data set mentioned.	
Number of episodes in collection(s)		
Number of episodes from the collection that appear in the publication	Number of episodes from the collection that appear in the publication rather than just the number of data fragments shown	

**Method**		
Methodological approach (as described by author(s))		
Reviewer’s comment on methodological approach (if relevant)		

FINDINGS OVERALL SUMMARY
Complete this table for each finding (complete for primary findings first and then secondary findings):

Summary of Findings		Notes
Phenomenon (in brief)		
Phenomenon in author's own words		
Research question/aim		
Number of relevant primary findings		
Number of relevant secondary findings		

RELEVANT INDIVIDUAL FINDINGS

**Findings**		**Notes**
Finding number	Number in the order they appear in the paper	
Phenomenon (in brief)		
Phenomenon in author's own words		
Research question for this finding (if applicable)		
Is talk about the future an inherent element or an incidental element of the phenomenon?		
Primary or secondary focus of research?		
Number of episodes pertaining to this finding	State number of episodes for this finding that appear in the article AND number of episodes for this finding in the collection (if information is available)	
Archetypal sequence		
Features of the talk in which the phenomenon is produced	Use I, II, III, IV, V, etc.	
What are the implications of these environmental features?	Why do it there?	
Sequence and/or turn design features of the phenomenon	Use A, B, C, D, E, etc.	
What are the interactional effects	Why do it like this?	

of these design features?		
In sum, what is the overarching function of the phenomenon?	Direct quote if possible	
Author-proposed implications		
Any other implications		
Notes		
